# Humans at the dawn of the in-body electrical nerve stimulation era

**DOI:** 10.52054/FVVO.14.4.041

**Published:** 2023-01-27

**Authors:** M Possover

**Affiliations:** Departments OBGYN, University of Cologne, Germany; Departments OBGYN, University of Aarhus, Denmark; Departments OBGYN, Jiaotong University, Xi’An, China

**Keywords:** Neuropelveology, Possover LION procedure, neuromodulation, spinal cord injured, in-body-ENS

## Abstract

**Background:**

The neuroprosthesis laparoscopic implantation technique for electric pelvic nerve stimulation was introduced to gynaecology over 15 years ago to treat intractable pelvic neuropathic pain. Following this first indication, other applications were developed, particularly in parapleology. The LION procedure developed to assist patients with paraplegia and common problems associated with inertia when confined to a wheelchair could find revolutionary applications in aging medicine and prevention.

**Material and Methods:**

Spinal cord injured patients who have undergone the Possover’s LION procedure.

**Main outcome measure:**

PubMed was systematically searched to identify peer-reviewed articles published in English that reported on LION procedure.

**Results:**

Three independent studies published recently (100 patients worldwide) have shown revolutionary recovery of supra-spinal control in patients with chronic spinal cord injury following pelvic nerves stimulation, with 70% of them establishing a walker/crutches-assisted gait. The same studies have also shown significant whole-body muscle-mass building, peripheral vasodilatation, and an unexpected improvement in bone mineral density.

**Conclusion:**

These ground-breaking findings could find revolutionary applications in aging medicine and the prevention of osteoporosis, with a huge impact on global public health. Humanity is on the cusp of an exciting new era following the introduction of the in-body electrical nerve stimulation technique.

**What's new?:**

In-body electrical nerve stimulation for recovery and/or control of human peripheral somatic and autonomic nervous systems.

## Introduction

### Treating functional body disorders in humans – transition from extracorporeal use to in-body implantation

Recent decades saw many new medical technologies developed to improve disorders or loss of body functions in humans, all finding their primary applications outside the human body. Thanks to the progress in medical materials and technologies, the development of sterilisation and antibiotherapy, many medical applications have undergone an unprecedented revolution, transiting from their primary applications outside the human body to in-body implantation.

In the first half of the nineteenth century, almost all bone fractures and dislocations were treated non-operatively. During the ensuing century, the operative treatment of fractures underwent an unprecedented revolution, first using external fixations, followed by the introduction into clinical practice of osteosynthesis including plates (Hansmann, 1886) and intramedullary nails (Seligson, 2015).

Vision disorders have been treated for centuries by magnifying loupes and glasses. Ophthalmology is evolving along a similar passage, applying technologies within the eye, including the implantation of innovative artificial or telescopic lenses.

Similarly, a century ago, hearing disorders were managed by the acoustic horn. In 1978, Rod Saunders, a 46-year-old hardware store manager, was fitted with a bionic ear in a laboratory at the University of Melbourne, Australia, designed by Graeme Clark. This bionic ear held a processor that transformed speech sounds into electrical stimulation to the inner ear. Since then, hearing implants have advanced considerably with the maturation of the technology and the rise of digital technology. However, reviving the hearing is just the beginning of what researchers hope to accomplish through rehabilitation by deploying artificial intelligence, robotics, and other approaches.

Invented in 1938 for patients with schizophrenia, electroconvulsive therapy consisted of administering a brief electric current to the frontal area of the brain to induce a generalised convulsive seizure. Electricity has been introduced into the human body this way ever since. Mahlon DeLong of Emory University and Alim Louis Benabid of Joseph Fourier University in France revealed how electrical stimulation of a different part of the nervous system could relieve symptoms of Parkinson’s disease. The treatment these researchers helped develop, known as deep-brain stimulation, uses electrodes implanted in the interior part of the brain to deliver electrical impulses to certain structures. Researchers would not have been able to develop cortical implants or deep-brain stimulation without fundamental knowledge about the brain’s organisation and function.

All this evolution reminds us of the “bionic man,” a science-fiction character from the 1970s. The Six Million Dollar Man told the adventures of Steve Austin, a former astronaut whose body was rebuilt with artificial parts after he escaped death. From science fiction, we come to reality. Intracerebral implants, psycho-dynamic cocktails, exoskeletons, or high-tech prostheses, humans - turned bionic - become augmented rather than repaired. Today, science is employing approaches such as artificial intelligence and robotics to delve deeper into the workings of the nervous system and restore abilities lost through injury or disease, including creating prosthetic arms that can feel.

### We are in a period of rapid progress for neuroscience: Would techno-scientific progress deprive us of the ambition to remain humans?

Today, researchers are extending works in neuroscience to deepen our knowledge of the brain and provide new options for patients. For example, scientists apply powerful artificial intelligence approaches to probe cognition and model how we think and perceive the world. Scientists are developing various prostheses that receive commands from the brain, allowing patients to control them. If someone has lost part of an extremity, an electronic device implanted in the remainder of the limb could read movement- related nerve signals from the brain and use them to operate a prosthetic extremity. Researchers have created brain implants that allow paralysed patients to employ their thoughts to move a prosthetic arm or even their immobile limbs. Early experiments have shown that researchers could provide paralysed patients with some control over their limbs by analysing their EEG patterns and transforming them into nerve control signals, enabling them to walk short distances, assisted by a walker and harness. Scientists are testing other applications for this type of mindreading brain-computer interface, potentially allowing people after a stroke or brain damage to communicate and improve rehabilitation therapy outcomes (Courtine and Sofroniew, 2019). Over the last 15 years, Possover has developed a revolutionary surgical technique: the laparoscopic implantation of neuroprosthesis, also knowns as the “LION procedure” (Possover, 2022b). The technique facilitates electrical stimulation to all pelvic-abdominal nerves and plexus following a minimally invasive procedure. This procedure has been developed for the treatment of intractable pelvic neuropathies (Possover et al., 2007) and pelvic organ dysfunctions (Possover et al., 2009). The most impressive indication of this technique is the implantation in people with spinal cord injuries for the recovery of some walking functions. Possover performed the first LION procedure in a paraplegic patient for the control of the bladder function and recovery of walking functions (Possover, 2009). In order to activate possible “redundant” spinal pathways and to lead to the capabilities for plasticity in the central nervous system, altering processes in the body to adapt (Filli and Schwab, 2015), Possover introduced the innovative concept of “continuous low-frequency pelvic nerves stimulation at 10Hz” in 2011, resulting in an antidromic stimulation of the low-motor neurons. Antidromic stimulation of motor axons recruits spinal Renshaw cells, which modulate motor neurons excitability and send collaterals to spinal locomotor circuits (Possover, 2021a; Alvarez and Fyffe, 2007; O’Donovan et al., 2010). However, in complete spinal paralysis, the fact that patients can only retrieve some “voluntary extension of the knees” when the pacemaker is “on” even on a minimal setting, suggests that the information signals might perhaps use not only nerve pathways but even unknown weak volitional motor input. Schneider showed that appropriate stimulation of axons away from the damaged area could lead to a normal passage of the action potential as a result of bypassing the demyelinated area and restoring its normal function, not just for a single axon, but also in the spinal cord (Schneider et al., 2010).

In a prospective study published 2021 on ten years’ experience with 31 spinal cord injured peoples, 71.4% of the patients were able to demonstrate an electrically assisted voluntary extension of the knee. 92.8% of the patients could get to their feet. 70% of treated individuals regained some volitional leg control and could walk >10 meters, showed reduced spasticity and increased voluntary stabilisation of the trunk (Possover, 2021a). Two further recent independent studies have shown similar recovery of supra-spinal control in patients with chronic spinal cord injury following pelvic nerve stimulation: they demonstrated 70% of patients with thoracic spinal cord injury and 60% of patients with cervical injury establishing a walker/crutches-assisted gait (Løve et al., 2021; Lemos et al., 2022; Possover, 2021b). Although the primary objective was to allow “robotic” walking by stimulating the muscles, continuous low-frequency stimulation of the pelvic nerves helped these patients recover the voluntary functions of the lower limbs and trunk necessary for walking.

Despite not being entirely certain how it works, the LION procedure for stimulation of the pelvic lumbosacral lower motor neurons already seems to provide a very feasible method, which allows people with SCI to stand up and to walk, knowing that almost every patient with an incomplete SCI can benefit from this procedure. Maximising the technique’s potential requires clarity on the still unclear mechanisms behind these functional recoveries (Possover 2021a)

### In-body electrical nerve stimulation (ENS) for aging control

According to 2006 U.S. Census Bureau data, the global demographic change through which we go now was predicted several years ago. The profound changes in the population age structure will have a major impact on life, health, and society. In this context, many discuss the aging society; there are more older people, life expectancy is increasing, and the number of younger people is decreasing. Since the effects of an aging society are already being felt, and the causes are not expected to change, social science forecasts predict that society will continue to age. By 2060, the older age group is predicted to increase by approximately 20 percent. Life expectancy also continues to rise thanks to scientific and medical advances. According to the Federal Statistical Office, the life expectancy by 2060 will be 84.4 years for newborn boys and as high as 88.1 years for girls. This prediction might not be reliable any longer since life expectancy in US has dropped considerably due to COVID-19, but in any case, the society is still getting older.

From around the age of 30, bone density decreases in both males and females. In females, this bone density loss accelerates after menopause. As a result, the bones become weaker and more brittle (osteoporosis), especially in old age. The aging process also affects the joints; cartilage and connective tissue changes can make the joint less resilient and more susceptible to injury. This process can lead to osteoarthritis, limiting joint mobility, often resulting in the need for prosthetic joint surgery. Muscle loss (sarcopenia) also begins around the age of 30 and continues through the remainder of one’s lifetime. The reported sarcopenia rates are 1–29% in community-dwelling populations and 14–33% in nursing home residents requiring long-term care (Cruz-Jentoft et al., 2014). Through this process, the muscle tissue and the number of muscle fibres gradually decrease, leading to increased stress on certain joints (e.g., knee and hip), resulting in arthritis, falls, functional loss of strength and balance capacity, and increasing gait hesitancy. These changes raise the risk of acute problems due to falls and injuries and chronic, recurrent, and degenerative illnesses (Ali and Garcia, 2014). Sarcopenia is responsible for considerable healthcare expenditure, with direct medical costs attributable to the disorder estimated at US$ 18.5 billion in 2000 in the United States (Janssen et al., 2004). Several therapies to prevent the aging process have been proposed, including mental activity, muscle training, and a high-protein diet. A crucial factor in this endeavour is to sustain high individual strength capacity and to do that, the muscle mass should be increased (Liu and Latham, 2009). Here, too, the introduction of in-body technology may change the performance of the human body. The in-body ENS may become a real option to slow down the aging process by preserving the body muscle mass. We have recently reported that following low-frequency stimulation of the pelvic nerves in patients with chronic spinal cord injury, the lower limb lean body mass increased exceptionally by an average of 32% (Possover, 2022a). In comparison, the classical Functional Electrical Stimulation- cycle ergometer showed an increase of 9.3% (Baldi et al., 1998). The new technologies developed to assist patients with paraplegia and common problems associated with inertia when confined to a wheelchair could find revolutionary applications in aging medicine and prevention. The in-body ENS could be suitable for older adults even more than patients with paraplegia, particularly those incapable of active muscle training because of pain, motoric limitations, or subcortical pathologies, and people confined to their bed for a long time (prophylaxis of decubitus!) (Possover, 2020). In-body ENS could open the door to a whole new area of human medicine, in which implanted electronics could help the human body perform better, resulting in longer life.

### Fitness training and bodybuilding through in-body ENS

One of the most ambitious applications of in-body ENS, even though it is more for medical prevention or quality of life than a purely medical application, is its introduction to the fitness and bodybuilding industry. Over three billion people are in workplaces. According to the Future of Wellness at Work study of the Global Wellness Institute, 76% of these people report struggling with their physical health. According to the International Health, Racquet & Sportsclub Association (IHRSA), the $30 billion health/fitness industry in the U.S. has been growing by at least 3-4% annually for the last ten years and shows no signs of slowing down any time soon. The IHRSA estimate suggested a total global fitness industry revenue of $94 billion in 2018.

The human body is a complex system consisting of many subsystems and regulatory pathways. The traditional idea that the cortex controls muscles in a one-to-one fashion has been challenged. In fact, functional interactions between supra- spinal, spinal, and peripheral regions can be integrated using network analysis as a common framework (d’Avella et al., 2003; Mussa-Ivaldi, 1999). Such a network theory can provide an alternative perspective on the modular organisation of the whole musculoskeletal system. The influence of biomechanics on functional muscle networks is expected to be most pronounced at lower frequencies: at low frequency (10Hz) connectivity increases within and between most leg muscles. This generates correlated activity at low frequencies that are fed back to spinal motor neurons via sensory afferents (Kerkman et al., 2018). This means practically that low frequency antidromic stimulation of the pelvic nerves, or even of only one single pelvic nerve such as the femoral nerve leads to activation and training not just of the stimulated muscles group but of all other muscle groups in the human body. Therefore, in- body ENS could become an extremely effective form of fitness training for those who do not have the time or opportunity to go to a gym.

Decreases in muscle activity and mechanical loading result in muscle atrophy, as was observed following spaceflights. Since the earliest days of human spaceflight, physiologists and NASA flight surgeons have recognised the importance of exercise to preserve the astronauts’ musculoskeletal system, including exercising for up to four hours in their daily routine. The cost of dedicating several hours a day in space for the sole purpose of exercising is very high. In-body ENS could help considerably reduce the exercise time required to maintain muscle mass in space.

### The in-body ENS of the human autonomic nervous system – an extraordinary, unexplored world

Nerve stimulation has been reserved until now for the central nervous system (spinal cord and deep brain stimulation) or the peripheral somatic nerves. The field of stimulating vegetative nerves and plexuses of the human body is practically unexplored. Because sympathetic innervation of the lower limbs originates in the lumbosacral plexus, the LION procedure enables stimulation of the sympathetic fibres of the lower extremities. If the observed effects of ENS on the muscle mass in patients with paraplegia were expected, the effects on blood circulation and bone mineral density were very surprising. Activation of the sympathetic nervous system classically induces peripheral vasoconstriction of resistance arteries, increasing cardiac activity, reducing the venous capacity, and decreasing bone mineral density. This is following the activation of alpha-1 adrenergic receptors by the norepinephrine released by post- ganglionic sympathetic neurons (Denise et al., 2009). However, recent clinical observations have shown exactly the opposite; plethysmographic examinations in patients with paraplegia treated by continuous low-frequency pelvic nerves stimulation have shown an unexpected explosive peripheral vasodilation as soon as the stimulus was turned on and an extraordinary regain of leg bone mineralisation – that would correspond to a nerve block and to an inactivation of the sympathetic pelvic nerves - or in contrary to an activation of the parasympathetic afferent pathways due to the antidromic stimulation of the low-motor neurons (Possover, 2022a). This phenomenon is supported by the findings of Muzquiz et al. (2021), who demonstrated that reversible low-frequency alternating current produced a high degree of nerve block at current levels comparable to the pulse stimulation amplitudes needed to activate nerves (Muzquiz et al., 2021). This discovery is of major interest since the application of the technique could have important consequences in preventing or even treating osteoporosis, a condition that is impacted by aging, causes over 8.9 million fractures annually worldwide (one fracture every 3 s) and affects 200 million women worldwide at a cost of US$ 13.8 billion/year (Stein and Wade, 2005). This phenomenon is also important for the Mars mission since it could help counter spaceflight-induced bone loss. From the perspective of the organism down to the lowest biological level, microgravity- induced osteoporosis differs from that encountered on earth. Microgravity appears to significantly alter the cellular cytoskeleton (Blaber et al., 2010) that, in an environment of near zero mechanical stress (alteration of the Wolff’s Law), is responsible systematically for osteoporosis. Osteoporosis due to microgravity causes the loss of calcified bony tissue at four times the rate on earth (around 2% per month). Furthermore, it does not appear to level off and appears to be much less reversible. Therefore, a three-year trip to Mars could potentially result in a devastating reduction of over 50% in bone mass (Axpe et al., 2020). Available countermeasures mainly consist of exercise and supplementation of bisphosphonate, however, these are not enough to maintain bone homeostasis. The resulting high systemic calcium levels could contribute to an increased risk of calcium stone formation. Kidney stones can usually be passed painfully, without surgery. Drinking plenty of water helps prevents the stones from forming and if present, to pass them. Urination in toilets in orbit is time-consuming; crewmembers are very busy, and urination is not an option if they do an extravehicular activity. Vomiting due to motion sickness can also lead to loss of fluids. Kidney stones could be the “final frontier” astronauts embarking on long missions would have to tackle. Much work is needed to find ways to prevent space travellers from developing kidney stones on long trips (Caillot-Augusseau et al., 1998). The low-frequency “in-body trainer” could help reduce periods of exercise considerably, may reduce the process of osteoporosis as in patients with paraplegia, and could act as a prophylactic treatment against kidney stone formation in microgravity.

## Conclusion

The autonomic nervous system is believed to regulate and control the entire body’s internal state, including many internal processes associated with the immune system and mental health. So, it is conceivable that in the future the in-body ENS will become an instrument of immunological therapy in oncology or in the treatment of psychological depression. Still, its neuromodulation effect on therapeutic and preventive medicine, and the huge impact on global public health seem unimaginable. Whether any of these new technologies and fields of investigation will have an impact similar to that of cochlear implants and deep-brain stimulation remains to be seen. We are on the cusp of an exciting new era: Humans are at the dawn of the in-body electrical nerve stimulation era!
